# Implementing Good Psychiatric Management in mental health services

**DOI:** 10.1177/10398562251378259

**Published:** 2025-09-12

**Authors:** Sikva Javaid, Sonja Quan, Lillian Ng, Rodrigo Ramalho, Lois Choi-Kain

**Affiliations:** 1415 The University of Auckland, Auckland, New Zealand; Health New Zealand Te Whatu Ora Te Toka Tumai, Auckland, New Zealand; 1415 The University of Auckland, Auckland, New Zealand; Health New Zealand Te Whatu Ora Te Toka Tumai, Auckland, New Zealand; 1415 The University of Auckland, Auckland, New Zealand; Gunderson Personality Disorders Institute, McLean Hospital, Belmont, MA, USA; Department of Psychiatry, Harvard Medical School, Boston, MA, USA

**Keywords:** Good Psychiatric Management, borderline personality disorder, mental health services, therapeutic alliance, psychoeducation

## Abstract

**Objective:**

Good Psychiatric Management (GPM) is a structured, evidence-based approach for treating borderline personality disorder (BPD). In this study, we aimed to explore the experiences of using GPM within a multidisciplinary mental health team and to identify factors that promote or impede its implementation and practice.

**Methods:**

The study design was informed by interpretive description methodology. Semi-structured, in-person interviews were conducted with staff based at an assertive community outreach service (ACOS) trained in GPM. Interviews were audio-recorded, transcribed and coded by reflexive thematic analysis.

**Results:**

From eleven participants of social work, community support work, nursing, psychology, occupational therapy and managerial backgrounds, we identified three main themes: (1) GPM as complementary to practitioners’ values, (2) GPM as empowerment to deliver treatment confidently and consistently and (3) leadership as instrumental for the implementation of a new model.

**Conclusions:**

Implementing GPM as a shared model of care has value for health professionals by improving confidence and skills in working with people with BPD and increasing team cohesion. Effective leadership facilitates the introduction of an empirically supported evidence-based model of care, even when there are system constraints. Further research is needed to evaluate the use of GPM in general healthcare settings.

Borderline personality disorder is common in mental health settings.^1^ Characteristic symptoms typically begin in late adolescence and include efforts to avoid abandonment, unstable and intense interpersonal relationships, an unstable self-image, impulsivity and recurrent suicidal behaviour and non-suicidal injury. BPD is diagnosed in adolescents and adults using the Diagnostic and Statistical Manual of Mental Disorders Text Revision (DSM-5-TR),^1^ although we acknowledge integration of dimensional constructs in diagnosing personality disorders.^2^ People with BPD are high users of health and social services and have a hospitalisation rate of 9 to 27% in emergency departments.^3^ Despite the development and identification of many manualised and specialised psychotherapies proven effective for BPD, mental health systems globally struggle to supply these treatments to meet the demand for care due to limited financial, human and material resources.^4^ Treating BPD in a constrained health system is further complicated when health professionals feel ill equipped to help.^4,5^

Gunderson’s model of GPM is a generalist approach that emphasises practical, interpersonal-focused care, combining elements of cognitive behavioural therapy and psychodynamic psychotherapy with a central formulation of BPD as a disorder of interpersonal hypersensitivity. GPM is founded on six principles to treat BPD^9^ that many providers already employ in psychiatric care (Table 1).^6^ GPM serves as a fundamental building block within stepped care that can upskill mental health professionals at every organisational level. If the patient does not respond adequately, the model is not necessarily abandoned. Rather, GPM can be utilised at all stages of treatment and combined with other treatment modalities. A stepped-care approach allows those with less severe symptoms to be managed with fewer resources, which increases the availability of resources and personnel for those who are in most need.^7^ GPM explicitly focuses on repairing personality problems by building an identity beyond treatment. In addition, GPM incorporates coherent approaches to diagnostic disclosure, psychoeducation, management of risk and safety planning, as well as the management of co-occurring disorders and medications.

**Table 1.** Principles of Good Psychiatric Management.

1. Be active (responsive, curious), not reactive
2. Support via listening, interest and selective validation
3. Focus on ‘getting a life’ – prioritise work and then relationships
4. The relationship is real (dyadic) and professional
5. Change is expected
6. Accountability – patients need to be active collaborators within treatment and assume control of their life

Adapted from ‘Good psychiatric management: a review’ by J. Gunderson et al.^30^ Copyright 2018 by Elsevier Ltd.

The efficacy of GPM was found comparable to Dialectical Behavioural Therapy (DBT) in one of the largest published methodologically rigorous randomised controlled trials of psychotherapies for BPD to date.^6,8^ At both the end of treatment and 2 years follow-up, GPM and DBT resulted in comparable declines in severe suicidal and non-suicidal self-injurious episodes,^6^ as well as improvements in a diverse range of outcomes including overall BPD symptoms, interpersonal functioning, anger, symptom distress, depression and anxiety. However, DBT treatment requires more intensive training compared to GPM, which has a less specialised approach and requires drastically fewer healthcare resources.

The comparable differences in outcomes of using DBT and GPM have important implications for health systems that provide and finance healthcare services, to promote, restore and maintain health.^9^ Inefficient systems have a negative impact on the quality, timeliness and accessibility of services and contribute to poorer outcomes.^10^ The Institute for Healthcare Improvement (IHI) supports healthcare organisations to optimise performance. The IHI’s framework of the Triple Aim uses a three-pronged approach, which seeks to improve health systems by targeting patient care experience, population health and reducing the per capita cost of health care.^11^ The emphasis on burnout among the healthcare workforce as a threat to patient-centredness^12^ led to addition of a fourth target (the Quadruple Aim) of improving healthcare workers’ experiences.^13–15^ Increasingly, health care requires interventions that adapt to changing conditions and understanding facilitating factors and barriers that embed or impede new models into clinical practice.^16^ The aim of this research was to explore the use of GPM, a pragmatic, effective generalist approach for the treatment of BPD by members of a multidisciplinary team working in a general adult mental health setting. We aimed to understand how GPM was applied in clinical interactions with patients with BPD and to explore its role in enhancing workers’ experiences in the community mental health context, in line with the Quadruple Aim framework as outlined above.

## Methods

### Research context and design

Participants were recruited from a publicly funded mental health service, an assertive community outreach service based at Health New Zealand Te Whatu Ora Te Toka Tumai. This service provides intensive community support for patients with mental illness, complex comorbidities, high psychosocial needs and frequent contact with the criminal justice system. The criteria for access to treatment are severe and recurring symptoms of psychotic and/or mood disorders, a consistent pattern of problematic engagement with services and/or treatment recommendations over an extended period of time, significant increased risks as a consequence of mental illness and two or more relapses of acute illness within the past 2 years.

In this study, interpretive description methodology was used, which acknowledges dual researcher–clinician influences on the research process and purposeful return of the findings to the field of practice.^17^ The researchers work across academia, clinical psychiatry and health systems. A reflexive approach was employed during recruitment, data collection and the analytic process of coding, interpreting and presenting findings.^18^

### Participants and recruitment

Participants were registered to complete the online Harvard Medical School GPM module^19^ and invited to take part in the study. Eleven individual interviews were conducted with staff.

The interview guide was piloted and tested with two junior doctors and refined iteratively after each interview, to enhance flow, streamline for relevance and minimise discrepancies apparent on initial questioning. The interview guide for managers emphasised organisational and administrative challenges of implementing GPM (Table 2). Written consent was obtained from all participants. In-person interviews were conducted in a private office and audio recorded. Interviews were transcribed verbatim and an anonymised copy was returned to participants.

**Table 2.** Interview schedule.

**1. How did you find the Good Psychiatric Management (GPM) training?**
What was most valuable about the training?
What would you change about the training?
What made the training difficult to undertake or fully complete?
**2. What do you think of the Good Psychiatric Management model?**
What aspects of GPM were most important to you?
How do you think practitioners in mental health care benefit from GPM?
How do you see yourself using GPM in your work?
What do you think of the Good Psychiatric Management model?
**3. How has GPM changed your understanding of whaiora?**
How has GPM affirmed how you engage with whaiora [service users]?
How has GPM challenged your previous ways of working?
**4. ** **D** **o you agree/disagree GPM is a model that could be used by mental health services?**
What would you change about GPM to make it better suited for working in New Zealand?
**5. What makes GPM a good fit for this health service, or otherwise?**
Looking ahead, how would you like to see GPM used within your team of practitioners?
What would help you develop competencies relating to GPM in the long run?
What makes it difficult for staff to use GPM in the long run?
**6. (For the leadership team) As someone on the management team at this health service, what was your experience of implementing GPM?**
What worked well during the implementation of GPM?
What would you change about the implementation process?
**7. (For the leadership team) What resources would be needed to support the implementation of GPM in the long run?**
How do you see yourself supporting the implementation of GPM in the long run?
What kind of leadership is needed to implement GPM?

### Analysis

Verbatim interview transcripts were deidentified and uploaded to NVivo (version 20.7.1). The transcripts were coded using reflexive thematic analysis (RTA)^20^: intensive familiarisation with the transcripts, coding of data using an inductive approach, documentation in Excel and NVivo, mind mapping of themes and diagrammatic representation using the online platform, Miro. Three research team members and a research intern independently co-coded portions of the transcripts. The codes were periodically reviewed and compared between research team members and discrepancies were resolved.

## Results

All eligible members of the team completed the full 8 hours of training except for one member of the team, and interviews were conducted with clinicians, allied health and social care practitioners and managers. Participants were of Māori, Pacific, Asian and European ethnicities, ranged in age from 37 to 62 years and had worked in their current position between 5 and 33 years (Table 3). Three main themes were identified: (1) GPM as complementary to practitioners’ values and health service needs, (2) GPM as empowerment to deliver treatment confidently and consistently and (3) leadership as instrumental for the implementation of a new model. In reporting results, the category of the participants’ profession is indicated using abbreviations: clinician (C), allied health or social worker (AS) or leadership position (L) (clinical coordinator or clinical team leader).

**Table 3.** Sample characteristics.

Profession	Length of time in position
Occupational therapist	13 years
Clinical coordinator	14 years
Community support worker	15 years
Nurse	33 years
Key worker/social worker	5 years
Nurse	20 years
Social worker	12 years
Psychologist	7 years
Community support worker	15 years
Clinical team leader	30 years
Social worker	21 years

### GPM as complementary to practitioners’ values

Participants found GPM acceptable and accessible. The GPM approach affirmed professional values of person-centredness, collaborative care and emphasis on social care. GPM was useful in upskilling teams in an efficient, effective and non-intensive way. GPM helped name existing practices and enhanced confidence clinical decision-making:

It didn’t feel foreign to me. It consolidated and affirmed the way I practice.

I hold [patients] with more empathy... I’m softer and more compassionate (AS).

Participants readily grasped the concepts of GPM:

You don't need years of experience or a degree…everybody can get this training to improve their skill level (C).

Some mentioned time constraints and limited facilities for training, for example, shared office space, yet GPM was *‘easy to use... you don’t need extra resources, you are the resource’ (L).* This practicality contributed to a collective upskilling of the team. Several participants suggested GPM principles could be adapted to use with Māori, Pacific and Asian ethnic groups.

### GPM as empowerment to deliver treatment confidently and consistently

Participants felt more confident in working with service users, by applying GPM principles in practice and having a common language to enhance team communication and cohesion.

It is empowering for me as a practitioner to feel I can implement psychological treatment on a daily basis with clients who have been judged to be the most difficult... I feel more confident and more empowered to do that (C).

Staff perceived they were more confident about working with their BPD clientele:

We’ve all done the same training, we’re all singing ideally from the same hymn sheet (L).

GPM provided clear guidance in collaborating with service users to enhance responsibility and agency:

Service users, they’re not getting pumped full of medications, they’re not getting sectioned and told to go to hospital, there’s an alternative way to work with people (AS).

It also challenged preconceptions about working with people with BPD:

There’s a fine line rescuing and helping a person out of a difficulty which is not necessarily clinically indicated or good for their own wellbeing (AS).

The whole concept of good enough is good… It takes away anxiety. I’ve always struggled working with people with borderline personality disorder... now I feel more confident (AS).

Also helpful was the explicit disclosure of a diagnosis of BPD in psychoeducation:

Giving the patient permission to decide whether they think they need to be under the mental health act or taking medication. It's a curious and open stance, it engages the person with some sense of responsibility for their own wellbeing (C).

### Leadership as instrumental implementation of a new model

Leadership and role-modelling helped sustain the use of GPM in the team setting:

[The lead clinician] brings it up all the time... advocates for it, always mentions GPM and points out GPM where it’s been used, should be used, how to use it, having someone in leadership, who believes in it, uses it, and talks about it regularly, keeps it at the forefront of your mind (L).

Participants appreciated GPM’s pragmatism and flexibility:

Free reign, to be able to do what we need to practice and make mistakes with support (AS).

## Discussion

In this study, we explored the experiences of a multidisciplinary team during the implementation of GPM in an assertive community outreach service. The results demonstrate how GPM can complement health professionals’ values, empower them to treat people (with or without a diagnosis of BPD) and the importance of leadership in role modelling and sustaining use of GPM. GPM incorporates common principles employed transdiagnostically in good clinical practice, which participants in our study confirm by noting familiarity with aspects of the approach. Our findings show that GPM is a feasible and pragmatic model for use in clinical practice. Participants identified with its proactive and reflective, rather than reactive, stance. Internationally, GPM is a standard practice for the treatment of BPD, allowing health professionals to undertake GPM training, regardless of expertise or experience.^21^ GPM can be used by a wide range of professions including counsellors, psychiatry residents, physicians, general practitioners and physician assistants.^22,23^ Training and using GPM enhanced understanding of their role and scope of evidence-based practice. GPM provided participants with practical skills to work with greater conviction and was particularly helpful for non-clinical professionals.

Clinicians expressed feeling more prepared and competent in directing patients to a more helpful care pathway. Developing more optimal formulations and care plans resulted in fewer patients remaining in need of services. Notably, the caseload of the team decreased by one-third following implementation of GPM. There was an improvement in staff morale as staff received the training. GPM dispels the myth that only staff who have receive specialist training can work with people who have BPD.

Participants reported feeling more confident in managing risk of non-suicidal self-injury and communicating within the multidisciplinary team, as GPM was relevantly integrated into regular team discussions.^24,25^ GPM was used to manage challenging situations by staff without the requirement for immediate escalation. The alignment of GPM strengthened participants’ belief in their role and the meaning of their work, rendering them better equipped to attend to patients’ needs and clarify their risks. The findings reflect a strong emphasis on leadership in introducing and embedding GPM to sustain its use. Professional development was a collective process of learning,^26^ where participants took responsibility for their training and were supported by managers and colleagues.^27^ GPM promoted an environment of collegiality which enhanced interprofessional collaboration. Alongside benefits for practice, we infer a sense of belonging, enhancing the experience of delivering care and potentially supporting workforce retention.^28^

This study emphasises hope as an important outcome of GPM training which mitigates the misconception among health professionals of BPD being untreatable. GPM training instils hope in health professionals and service users alike. GPM encourages professionals to establish and maintain boundaries and better communicate to enhance the therapeutic alliance. Furthermore, this study supports the notion of GPM as a general model that healthcare professionals can use for other psychiatric disorders. The GPM approach is considered safe, pragmatic and effective. However, as with other psychological approaches, there can be adverse effects. Potential risks arising from the implementation of GPM with limited staff training include worsening of symptoms, self-harm and interpersonal problems. GPM training emphasises reflective practice in holding risks related to distress associated with BPD. These risks are mitigated when GPM is delivered collaboratively by clinicians, with attention paid to the therapeutic relationship and patient agency.

## Limitations of the study and directions for future research

This study addresses a gap in the literature in exploring the experiential implementation of GPM using qualitative methodology in one assertive community outreach service that attends to the needs of people with severe mental illnesses and high psychosocial needs. GPM has a limited evidence-based approach with just one randomised control trial that supports its clinical effectiveness. Therefore, we cautiously interpret our study findings in recommending implementation of GPM which may be less transferable to other contexts because of the specialised nature of the service and the small sample size. We refer to Malterud’s concept of information power, whereby the criteria for quality rest on the applied methodology and robustness of the analysis.^29^ A further limitation of this study is that there was no minimum requirement of course hours to complete the 8-h Harvard Medical School GPM online module.^19^ Participants who did not complete the full training may have had more limited experience of using GPM in practice. This may have been mitigated by the influence of the lead clinician who encouraged staff learn and practice GPM. Thus, we acknowledge affiliation bias as a potential limitation. One author was affiliated with the health context in which the research was conducted. Their involvement in the research process was minimal but may have influenced participants’ perspectives of GPM.

We recommend further research in community mental health and in general healthcare settings, to test the use and applicability of GPM by other health professionals in working with people who have BPD, and in particular, to evaluate the cultural acceptability of GPM for indigenous and minority ethnic groups.

## Conclusion

Implementing GPM as a shared model of care has value for mental healthcare clinicians and enhances communication and collaboration within teams. GPM increases staff confidence and skills, enabling them to work more compassionately with people with BPD. Amidst system constraints, psychiatric services can support work-based learning with effective leadership to help implement an empirically validated model of care. Further research is needed to evaluate the use of GPM in general healthcare settings.
